# Planning of Green Space Ecological Network in Urban Areas: An Example of Nanchang, China

**DOI:** 10.3390/ijerph121012889

**Published:** 2015-10-15

**Authors:** Haifeng Li, Wenbo Chen, Wei He

**Affiliations:** 1 College of Land Resource and Environment, Jiangxi Agricultural University, Nanchang 330045, China; E-Mails: haifengl1984@jxau.edu.cn (H.L.); hewei0703611@163.com (W.H.); 2 Key Laboratory of Landscape and Environment, Nanchang 330045, China; 3 College of City and Tourism, Hengyang Normal University, Hengyang 421002, China

**Keywords:** green space, ecological network, landscape pattern analysis, network structure analysis, urban area

## Abstract

Green space plays an important role in sustainable urban development and ecology by virtue of multiple environmental, recreational, and economic benefits. Constructing an effective and harmonious urban ecological network and maintaining a sustainable living environment in response to rapid urbanization are the key issues required to be resolved by landscape planners. In this paper, Nanchang City, China was selected as a study area. Based on a series of landscape metrics, the landscape pattern analysis of the current (in 2005) and planned (in 2020) green space system were, respectively, conducted by using FRAGSTATS 3.3 software. Considering the actual situation of the Nanchang urban area, a “one river and two banks, north and south twin cities” ecological network was constructed by using network analysis. Moreover, the ecological network was assessed by using corridor structure analysis, and the improvement of an ecological network on the urban landscape was quantitatively assessed through a comparison between the ecological network and green space system planning. The results indicated that: (1) compared to the green space system in 2005, the planned green space system in 2020 of the Nanchang urban area will decline in both districts (Changnan and Changbei districts). Meanwhile, an increase in patch density and a decrease in mean patch size of green space patches at the landscape level implies the fragmentation of the urban green space landscape. In other words, the planned green space system does not necessarily improve the present green space system; (2) the ecological network of two districts has high corridor density, while Changnan’s ecological network has higher connectivity, but Changbei’s ecological network is more viable from an economic point of view, since it has relatively higher cost efficiency; (3) decrease in patch density, Euclidean nearest neighbor distance, and an increase in mean patch size and connectivity implied that the ecological network could improve landscape connectivity greatly, as compared with the planned green space system. That is to say, the planned ecological network would reduce landscape fragmentation, and increase the shape complexity of green space patches and landscape connectivity. As a result, the quality of the urban ecological environment would be improved.

## 1. Introduction

Urbanization and industrialization can be viewed as essential causes for land use and land cover change, and it also increases with the majority of the world’s population migrating into cities [1,2]. China’s urban population in 2001 accounted for 37.7% of its total population, and this proportion may reach 75% in 2050 by estimation [3]. However, rapid urbanization, accompanied by the marked rise of the human population, has caused many environmental impacts associated with the reduction of green spaces [4,5,6]. Urban environments are increasingly exposed to severe conditions of thermal stress, air pollution, and noise nuisances [7,8], which critically influence the health and well-being of the cities’ inhabitants [9]. It appears from a review of the relevant literature that the air quality in China has become an increasing public concern because of its health risks [10].

Green spaces, a component of ecosystems, play an important role in complex urban ecosystems [11] and provide significant ecosystem services with environmental, aesthetic, recreational and economic benefits [12,13,14]. Urban green spaces moderate the impact of the negative consequences of human activities by, for example, absorbing pollutants and releasing oxygen [15], moderating or buffering the effects of noise [16], offset or mitigate the urban heat island, and conserve energy [17,18,19,20], *etc*. Moreover, green spaces are now widely viewed as a health-promoting characteristic of residential environments [21,22], and have been linked to mental health benefits, such as recovery from mental fatigue and reduced stress [23,24]. According to the World Health Organization the living conditions in the urban environment are the key to the health and well-being of its inhabitants [25]. Recognition of the importance of green space in urban ecosystems has led to considerable work on urban green space planning to improve the urban environment and enhance the quality of life [6,26].

As the capital city of Jiangxi province, Nanchang is now the fastest growing area among all major cities in the middle-and-lower reaches of the Yangtze River. In recent years, there has been a substantial amount of infrastructure improvement, such as ongoing subway construction, numerous demolition projects of older buildings, and construction of new buildings. Unfortunately, green space preservation is usually ignored or underestimated by urban planning policy-makers and builders, with the result that remnant urban green spaces are being gradually encroached upon and fragmented by urban sprawl. Furthermore, the decline and fragmentation of urban green spaces, combined with an increasing number of motor vehicles on the roads, have led to the increased level of air pollution, which results in subsequent negative effects on human health [27]. Therefore, how to conserve and create urban green spaces in response to rapid urbanization is the key issue needed to be resolved by urban landscape planners in Nanchang City. Recent research shows that green space networks can provide a solution to the problems of intensified land use and fragmentation [4]. Green space networks refer to the configuration and management of green space as a functional system [28]. Development of green space networks is increasingly considered a suitable approach to improve the ecological value of urban green space and the urban environment [29,30,31,32]. Moreover, various methods and principles of landscape ecology have been applied to green space ecological network planning, such as landscape pattern metrics [13,33] and network analysis [34]. However, less attention has been paid to the method integrating landscape metrics with network analysis in the planning of urban green space ecological networks [35]. In this study, network analysis has been integrated with landscape pattern metrics for the assessment and the optimization of an urban green space system plan. The main purpose of this study is to construct a green space ecological network in an urban area and to assess whether or not the network would improve the quality of the green space of the urban area.

## 2. Materials and Methods

### 2.1. Study Area

This study was conducted in Nanchang City (28°09′ N–29°11′ N, 115°27′ E–116°35′ E), the capital of Jiangxi Province in China. It is located on the southwest bank of Poyang Lake and it lies in the middle and lower reaches of the Yangtze River. This area belongs to a subtropical zone with a humid monsoon climate. Nanchang City has a temperate climate with well-marked seasons, plenty of rainfall and sunshine, with an average temperature of 17–17.7 °C and annual precipitation of 1600–1700 mm. The city has experienced dramatic population growth (from 1.34 million in 1990 to 5.04 million in 2010) [27] and has sprawled greatly in the last 50 years (617 km^2^).

### 2.2. Data Sources

**Figure 1:**
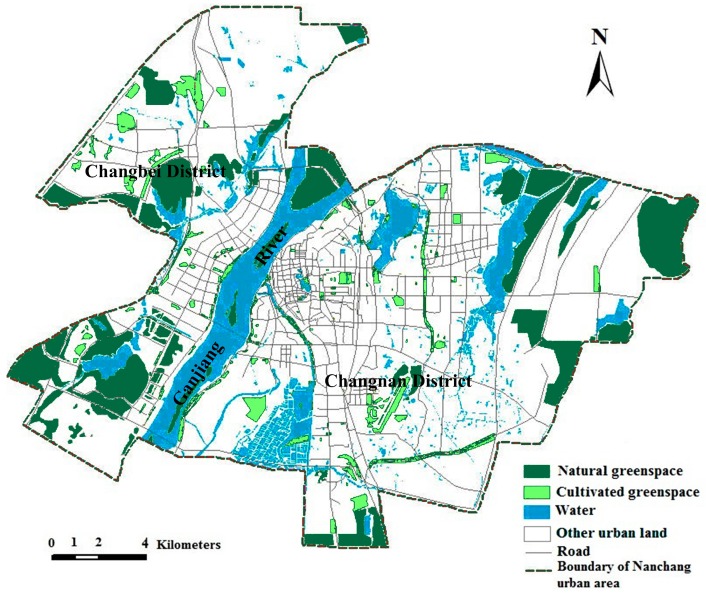
Distribution map of urban green space in 2005.

**Figure 2:**
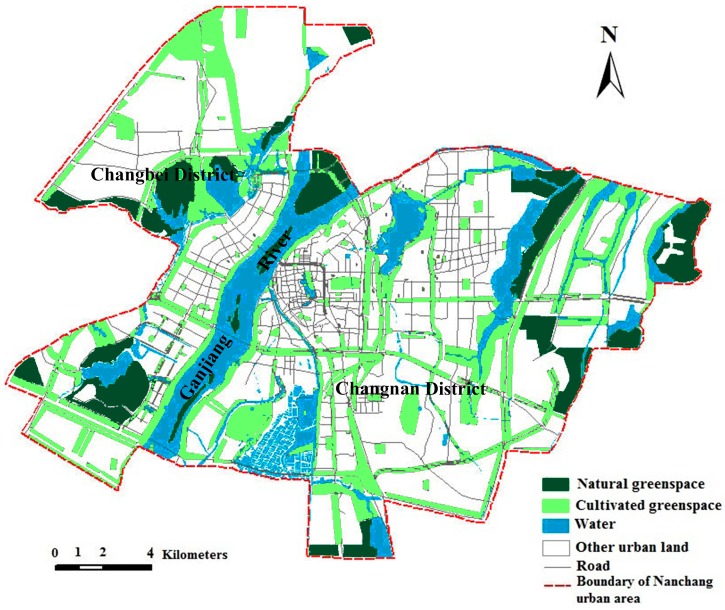
Urban green space system planning map of Nanchang in 2020.

A green spaces distribution map was used for landscape pattern analysis. An urban green space system planning map, present layout of urban traffic, and an urban traffic planning map of Nanchang City (2003–2020) were also used. Green spaces were classified into two types: natural green space and cultivated green space. Eco-green space, woodland for landscape, parts of green space of waterfront, and parts of shelter greenbelt have been integrated into natural green space. Thereafter, public green space, green land for production, green space, garden, greenbelt pertaining to workplace, and the rest of shelter greenbelt have been amalgamated into cultivated green space. The natural green space is defined as land that has been designated to remain in a predominantly open, natural, and undeveloped state with semi-natural plant communities, including mainly woodlands and semi-natural hill vegetation and cultivated green space [35]. The cultivated green space includes habitats, such as urban parks, gardens, and street green spaces. This simplified land use classification scheme was adopted for the purpose of assessing the spatial characteristics of the major human modifications to natural or semi-natural landscapes, and for understanding the general patterns of their interactions. The vector data of the green space system in 2005 and a green space plan in 2020 of the Nanchang urban area (Figure 1 and Figure 2) were converted to raster format at pixels of 10 m × 10 m, by using Convention Tools of ArcGIS 9.3. The Nanchang urban area was demarcated by the Ganjiang River and has been resolved into Changbei district and Changnan district to construct the ecological network.

### 2.3. Analysis of Landscape Pattern

Landscape pattern indices promote landscape pattern analysis from a qualitative analysis to a quantitative analysis, and they are widely used to analyze the characteristics of landscape patterns [2]. The landscape pattern characteristics can be analyzed on different levels (*i.e.*, individual patch level, class level, and landscape level) by using the public domain version of FRAGSTATS [36]; therefore, the version 3.3 of FRAGSTATS was used in this study. A small subset of the landscape pattern metrics which had specific ecological significance was selected to assess the green space system for the Nanchang urban area. It includes compositional and configurational metrics, *i.e.*, total class area (CA), patch density (PD), edge density (ED), mean patch size (MPS), Shannon’s diversity index (SHDI), Shannon’s evenness index (SHEI), landscape shape index (LSI), mean patch fractal dimension (MPFD), Euclidean nearest neighbor distance (ENN), and connectivity (CONNECT). The selected metrics were numerically reliable (*i.e.*, showing consistent trends) for depicting landscape patterns [35]. They are defined as follows:

(1) $$\text{CA = }\sum_{\text{j=1}}^{\text{a}}{\text{a}}_{\text{ij}}(\frac{1}{10000})$$

where a*_ij_* means the area of patches (m^2^), the units of CA is hectares, CA > 0, no limit. It is a measure of landscape composition.

(2) $$\text{PD = }\frac{\text{N}}{\text{A}}\;\times \;1000000$$

where N means the total number of patches and A is the total landscape area. The unit of PD is the number per 100 hectares, PD > 0, constrained by cell size. It is a simple measure of the fragmentation of the patch type.

(3) $$\text{ED = }\frac{\text{E}}{\text{A}}\;\times \;10000$$

where E is the sum of the lengths (m) of all edge segments in the landscape and A is the total area of the landscape. Edge density (ED) reflects the ratio of landscape circumference to the landscape area, which is expressed in m/ha [37].

(4) $$\text{MPS = }\frac{\text{A}}{\text{N}}(\frac{1}{10000})$$

where A is the total area of the landscape and N means the total number of patches. It is a simple measure of the fragmentation of the patch type [32].

(5) $$\text{SHDI = }-\sum_{\text{k=1}}^{\text{n}}{\text{P}}_{\text{k}}\text{ln}（{\text{P}}_{\text{k}}）$$

(6) $$\text{SHEI = }\frac{\text{SHDI}}{{\text{SHDI}}_{\text{max}}}\text{ = }\frac{-\sum_{\text{k=1}}^{\text{n}}{\text{P}}_{\text{k}}\text{ln}（{\text{P}}_{\text{k}}）}{\ln（\text{n}）}$$

where P_k_ is the proportional of each patch type in landscape and n is the number of patch types. Shannon’s diversity index is a measure of patch diversity in a landscape that is determined by both the number of different patch types and the proportional distribution of area among patch types. Shannon’s evenness index is the ratio of the actual value of the Shannon diversity to its maximum value that is achieved when all patch types each occupy the same proportion of the landscape [2,38].

(7) $$\text{LSI = }\frac{\text{0.25E}}{\sqrt{\text{A}}}$$

where E is the total length of patch edges and A is the total area of the landscape,a standardized measure of patch compactness that adjusts for the size of the patch. As LSI increases, the patches become increasingly dis-aggregated.

(8) $$\text{MPFD = }\frac{\sum_{\text{i=1}}^{\text{m}}\sum_{\text{j}=1}^{\text{n}}\left[\frac{2\ln\left(0.25{\text{P}}_{\text{ij}}\right)}{\ln\left({\text{a}}_{\text{ij}}\right)}\right]}{\text{N}}$$

where P_ij_ is the length of patches and a_ij_ is the area of the patches.

Euclidean nearest neighbor distance (ENN) equals the distance (m) mean value over all urban green space patches to the nearest neighboring patch, based on the shortest edge-to-edge distance from cell center to cell center [36,39].

(9) $$\text{CONNECT = }\left[\frac{\sum_{\text{i}=1}^{m}\sum_{j=k}^{n}c_{ijk}}{\sum_{i=1}^{m}\left(\frac{n_{i}\left(n_{i}-1\right)}{2}\right)}\right]\left(100\right)$$

where c*_ijk_* = 0 if patches *j* and *k* are not within the specified distance of each other and c*_ijk_* = 1 if patches *j* and *k* are within the specified distance.

### 2.4. Network Analysis

Network structure analysis introduces a process for aggregating results of patch and corridor (line) analysis and incorporates indicators that describe interrelationships between landscape elements. The graph theory analyzes networks to optimize a given flow-related objective [40]. Landscape ecologists have used the theory to reduce complex landscapes into understandable spatial configurations and uncover flow patterns [41]. The graph theory redefines complex systems as a finite set of nodes and linkages, and uses rules to define which edges join which pairs of nodes. The networks in the present study were described in this manner using green space patches as nodes and corridors as links [32].

The number, length, and density of corridors were undertaken to describe their structural characteristics. The complexity of a network can be measured by the concepts of network circuitry, node/line ratio, network connectivity, and cost ratio [29]. Network circuitry is interpreted as the degree to which loops are present in the network. It can be measured by α index, *i.e*., the number of loops present divided by the maximum number of loops possible:

(10) $$\text{α = }\frac{l\;-\;v\;+\;1}{2v\;-\;5}$$

where *l* is the number of corridors and *v* is the number of nodes. The α index ranges from 0, for a network with no loops, to 1.0 for a network with the maximum possible number of loops present [42].

Node/line ratio can be measured by β index, is calculated by following formula:

(11) $$\text{β = }\frac{l}{v}$$

where *l* is the number of corridors and *v* is the number of nodes. When β is smaller than one, it means that the network takes on a dendroid pattern. When β is equal to one, it means that there is single loop in the network. When β is greater than one, it means that there is more complex connectivity in the network [43].

Network connectivity can be measured by the γ index, the ratio of the number of links in a network to the maximum number of links possible:

(12) $$\text{γ = }\frac{l}{l_{\max}}\;=\;\frac{l}{3(v-2)}$$

The γ index varies from 0, indicating that none of the nodes are linked, to 1.0, where every node is linked to every other possible node [42].

The cost ratio is based on the landscape conditions and socio-economic realities and is calculated by following formula:

(13) Cost Ratio = 1−(l/d)

where *l* is the number of corridors and *d* is the length of corridors [44]. Cost ratio is a measure of efficiency and will inevitably have to return to concrete measures to account for cost differentials of alternative green space networks [40].

### 2.5. Green Space Ecological Network Delineation

**Figure 3:**
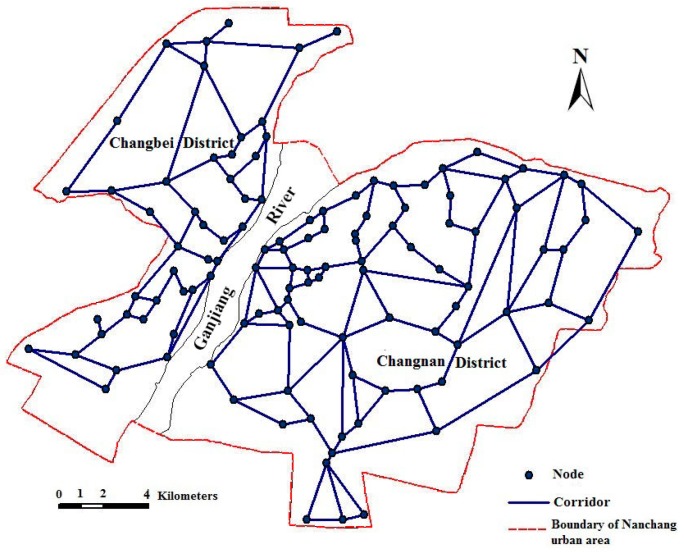
Distribution of ecological nodes and corridors of the Nanchang urban area.

**Figure 4:**
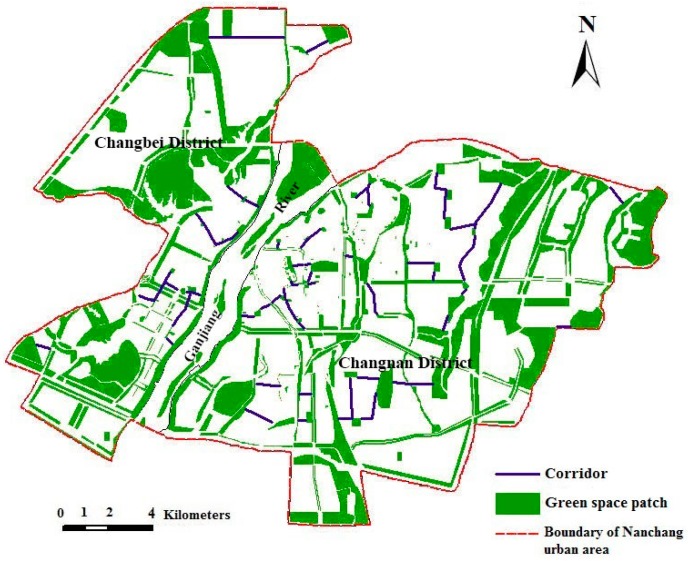
Planning of ecological network for the Nanchang urban area.

Within the green space planning framework, nodes refer to any discrete non-linear patch, while links are linear elements that are to be included in the ecological network plan. In other words, a planned ecological network is a graph consisting of patches and corridors [35]. The evaluation of green space networks includes analyses of patch and corridor characteristics and the connectivity [29,32]. The degrees of connectivity become indices for linkage of network elements analysis. Several indices have been developed for this purpose [45,46]. In this study, corridor density and corridor length were used for analysis, comparing features in 2005 with the planned results in 2020. The improvement of ecological networks on urban landscape was quantitatively assessed through a comparison between the ecological network planning and green space system plan (in 2020) for the Nanchang urban area. It is reported that a greenbelt with the width more than 30 m should be built against noise [47]. In this study, the corridors in the ecological network planning (Figure 3) were normalized and have been regarded as green space, the width of the corridors was assumed as 40 m. These corridors were subsequently overlaid on the urban green space system planning map, while the land use types, in addition, were combined into a single type, *i.e.*, non-green fields (Figure 4). Furthermore, landscape metrics on the landscape level for the ecological network planning were calculated.

## 3. Results

### 3.1. Landscape Pattern Analysis with Class-Level Metrics

The result of landscape pattern analysis with class-level metrics was shown in Table 1.

**Table 1:** The comparison of landscape metrics on class level.

Landscape Metrics	Changbei District				Changnan District			
	Natural Green Space		Cultivated Green Space		Natural Green Space		Cultivated Green Space	
	Status in 2005	Planning in 2020	Status in 2005	Planning in 2020	Status in 2005	Planning in 2020	Status in 2005	Status in 2005
CA (ha)	2286.76	1361.06	498.79	2594.79	2184.95	1654.94	842.40	4227.40
PD (number per 100 hectares)	0.35	0.09	0.65	1.08	0.09	0.35	1.08	0.99
ED (m·m^−2^)	14.05	7.03	9.33	30.29	5.07	5.09	10.36	32.02
MPS (ha)	51.97	123.73	6.16	19.22	115.00	21.49	3.57	19.66
MPFD	1.08	1.09	1.09	1.10	1.07	1.07	1.08	1.10

Comparing the green space system in 2005 with the green space planning in 2020, natural green space will decline while cultivated green space will be expanded in both districts after implementing the planning. The total area of cultivated green space will increase by 2096 ha in Changbei district and 3385 ha in Changnan district, respectively. At the end of the planning period, the total area of green space will experience a marked increase.

In Changbei district, the patch density of cultivated green space will increase by 0.43 per 100 hectares, but reduce by 0.26 per 100 hectares for natural green space. The edge density and the mean patch fractal dimension remain nearly unchanged. However, the mean patch size and the mean patch fractal dimension of natural green space and cultivated green space will both be increased.

In Changnan district, the patch density of cultivated green space and natural green space changes in the opposite direction compared to Changbei district. The edge density natural green space and cultivated green space will both be increased. The mean patch size of natural green space and cultivated green space will all increase. The mean patch fractal dimension of natural green space has nearly no change, but will increases for cultivated green space.

### 3.2. Landscape Pattern Analysis with Landscape-Level Metrics

To capture the synoptic characteristics of the green space system in 2005 and green space planning in 2020, the natural and cultivated green space patch types were merged into one green space patch type. The result of landscape pattern analysis with landscape-level metrics is presented in Table 2.

In Changbei district, the patch density does not show any changes. Meanwhile, the edge density, the mean patch size, the mean patch fractal dimension, the landscape shape index, the connectivity, the Shannon’s diversity index, and Shannon’s evenness index will all increase. However, the Euclidean nearest neighbor distance decrease from 88.82 m to 49.39 m.

In Changnan district, the patch density, the edge density, the landscape shape index, the connectivity, the Shannon’s diversity index, and Shannon’s evenness index increase. However, the mean patch fractal and the Euclidean nearest neighbor distance both decrease. Meanwhile, the mean patch fractal dimension has nearly no change.

**Table 2:** Landscape metrics on landscape level.

Landscape Metrics	Changbei District		Changnan District	
	Status in 2005	Planning in 2020	Status in 2005	Planning in 2020
PD (number per 100 hectares)	1.85	1.85	1.47	2.41
ED (m·ha^−1^)	23.37	36.04	15.43	36.41
MPS (ha)	53.92	54.03	67.99	41.57
LSI	8.47	12.01	7.14	14.89
SHDI	0.53	0.63	0.40	0.58
SHEI	0.77	0.90	0.58	0.84
MPFD	1.06	1.07	1.07	1.07
ENN(m)	88.82	49.39	100.58	43.68
CONNECT	4.68	4.79	2.76	3.05

### 3.3. General Evaluation of Green Space Networks

The green space patches with great ecological and historical human importance were selected and abstracted as ecological nodes, such as Diezi Lake, Xianghu Lake, Bayi Square, *etc*. In this study, 118 ecological nodes were selected in Nanchang urban area, in which 44 nodes were in Changbei District and 74 in Changnan District (Figure 3). According to the construction principles of ecological networks, buildings and roads are serious barriers to connectivity and serve to isolate green space; therefore, they should be avoided in the construction of ecological corridors. Moreover, duplicated links (corridors) between each pair of nodes of network should also be avoided. In the present study, 165 corridors were constructed, in which 56 corridors were in Changbei District and 109 in Changnan District (Figure 3). Then, the ecological network of the Nanchang urban area was built based on graph theory. However, as street green spaces and rivers in the urban landscape could be integrated with the ecological network as corridors [35,48], some links were modified from linear to green space forms that follow the roads and rivers to make the links feasible based on graph theory using the urban traffic plan of Nanchang City (2003–2020). Finally, a “one river, two banks, north and south twin cities” planning of ecological networks for the Nanchang urban area was proposed (Figure 4).

Using corridor structure analysis and network structure analysis to assess the planning ecological network, the results show that the ecological network of the two districts having high corridor density, of 0.81 km/km^2^ in Changbei and 0.90 km/km^2^ in Changnan, respectively. The α index, β index, γ index, and cost ratio of the ecological network in Changnan is higher than Changbei, which indicates that the ecological network of Changnan has higher connectivity, and Changbei’s ecological network has relatively higher cost efficiency and is more viable from an economic point of view (Table 3).

**Table 3:** The corridor structure metrics for the ecological network planning.

	Nodes	Corridor Number	Corridor Length (km)	Corridor Density (km·km^−2^)	α Index	β Index	γ Index	Cost Ratio
Changbei District	44	56	100.45	0.81	0.23	1.40	0.49	0.44
Changnan District	74	109	197.15	0.90	0.25	1.47	0.50	0.45

Compared to the planned green space system in 2020, landscape metrics of the ecological network planning in both districts of the Nanchang urban area has significantly changed (Table 4). In Changbei district, the patch density decreases from 1.85 per 100 hectares to 1.46 per 100 hectares, and the Euclidean nearest neighbor distance decreases from 49.39 m to 40.01 m. However, the mean patch size, the landscape shape index, the mean patch fractal dimension, the connectivity, the Shannon’s diversity index, and Shannon’s evenness index have all increased.

In Changnan district, the patch density decreased from 2.41 per 100 hectares to 1.57 per 100 hectares, and the Euclidean nearest neighbor distance decreased from 43.68 m to 38.45 m. Instead, the mean patch size, the landscape shape index, the mean patch fractal dimension, the connectivity, the Shannon’s diversity index, and Shannon’s evenness index have all increased.

**Table 4:** Landscape metrics in different plans.

Landscape Metrics	Changbei District		Changnan District	
	Green Space System Plan in 2020	Ecological Network Planning	Green Space System Plan in 2020	Ecological Network Planning
PD (number per 100 hectares)	1.85	1.46	2.41	1.57
ED (m·ha^−1^)	36.04	40.52	36.41	41.23
MPS (ha)	54.03	60.13	41.57	45.23
SHDI	0.63	0.78	0.58	0.76
SHEI	0.90	0.99	0.84	0.95
LSI	12.01	14.15	14.89	15.78
MPFD	1.07	1.08	1.07	1.08
ENN(m)	49.39	40.01	43.68	38.45
CONNECT	4.79	5.13	3.05	3.56

## 4. Discussion

Green space plays an irreplaceable role in the healthy maintenance of urban ecosystems [49], while also meeting social and psychological needs of the urban population [50,51]. However, the lack of effective and comprehensive planning has caused uncontrolled urban expansion; consequently, many natural green spaces will be converted to construction land. The inevitable occupation of natural greenbelts in urban development processes could be the cause of the reduction of the natural green space. Nevertheless, this study showed that cultivated green space and the total class area of green space patches should be expanded in both districts of the Nanchang urban area if the green space system planning gets started. This will offset the loss of natural green space due to the urban area construction. In this sense, the green space system planning could improve the present green space system. As noted earlier, similar results were found by assessing the recent greenway augmentation plan of Xianmen Island [35].

In this paper, opposite results were discovered in the patch density and mean patch size changes of cultivated green space and natural green space between two districts of the Nanchang urban area at the class level. A possible explanation for this is that the smaller natural green space patches have been occupied while larger patches will be preserved in the process of urban construction, the additional cultivated green space patches have a larger area, and the smaller patches will be aggregated into larger patches in green space system planning. Consequently, fragmentation of the natural green space patches in Changbei district and cultivated green space patches in Changnan district have an apparently descending tendency. On the contrary, this study has also demonstrated that the patch density should increase at the landscape level in Changnan district. It indicates that the landscape should be highly fragmented [52]. Fragmentation of urban green space not only reduces the health of urbanized ecosystems [53], but it also deteriorates the quality of living and working environments, threatening urban sustainability, especially in congested cities [54]. In this regard, green space system planning does not necessarily improve the present green space system because the fragmentation of urban green space landscape increased. Nonetheless, the planned green space system in 2020 could improve landscape diversity, embodied with increases in connectivity, Shannon’s diversity index, and a decrease in Euclidean nearest neighbor distance. Previous studies have indicated that the spatial pattern of urban green spaces could be improved by the following measures: making the green spaces more natural with diverse vegetation configurations [55] and complicated shapes, and connect the green spaces to form green networks with multifunctional greenways and green corridors [33,56] to enhance proximity and ecosystem services to both nature and people [57]. Therefore, green space system planning should always be conducted because it plays a crucial role in the process of urban development.

The planning of the urban green space ecological network is mainly a reaction to urban sprawl and the dramatic loss of natural areas [50]. It includes protection of existing green spaces, creation of new spatial forms, and restoration and maintenance of connectivity among diverse green spaces [32]. In this study, a decrease in patch density on the landscape was found in both districts of the Nanchang urban area, which implies that the landscape fragmentation should decline. The decreases in Euclidean nearest neighbor distance and increase in mean patch size and connectivity indicates that the ecological network could enhance landscape connectivity greatly. Similar studies have shown that the higher connectivity the ecological network has, the more favorable it is to the matter cycle and energy flux [35,46]. Overall, the results revealed that the planning of a green space ecological network could further improve the quantity and quality of the present green space system of the Nanchang urban area considerably, and the green space patches could become larger, geometrically more complex, and be ecologically more connected and diverse. Highlighting the role of a green space ecological network is of particular importance for urban green space system planning.

## 5. Conclusions

The green space ecological network comprised of two districts, both with high corridor density, in the Nanchang urban area. Planning an ecological network would reduce landscape fragmentation, and increase the shape complexity of green space patches and landscape connectivity. Planning of green space ecological networks could further improve the quantity and quality of the green space system of the Nanchang urban area considerably. As a result, the quality of the urban ecological environment would be improved.

Although apparently conclusive, the results of this research on planning green space ecological networks have limitations and further research is warranted. One important point is that this study just focuses on the current situation of that of the Nanchang urban area. demarcated by the Ganjiang River and the river becomes an insurmountable obstacle for migration of terricolous biology beside the river. Though the migration should be completed by greening the bridge over the river as a green space path, this choice has been abandoned because it is expensive, resulting in possible bad effects to the terricolous biology and traffic. Consequently, a “one river and two banks, north and south twin cities” ecological network planning has been introduced, in which the ecological nodes are abstracted from green space patches with great ecological and historical human importance, and the water in the urban area is used fully as ecological corridors. In addition, the green space system planning (in 2020) is excessively influenced by overall city plans in recent years; thus, the effect of green space system planning has be limited to some extent. Fortunately, the green space ecological network planning in this study has compensated for these defects. However, the ecological network planning is a system of linear networks, and it cannot replace the overall urban planning. Revealing the relationship between urban green space ecological network planning and overall urban planning would help green space ecological networks perform their functions adequately, and even be useful for the construction of comprehensive green space plans. Finally, this research was conducted for one city; the results of green space pattern changes brought by ecological network planning may differ for other cities. Thus, further research is needed to compare in order to validate the conclusions.
